# Effectiveness of video assisted teaching program for caregivers on gross motor outcome among children with cerebral palsy undergoing hyperbaric oxygen therapy: A quasi-experimental study

**DOI:** 10.1038/s41598-025-14038-4

**Published:** 2025-08-05

**Authors:** Rania Ebrahim Abd-Elzaher, Ebtisam Mohamed Abd-El-Aal, Amina Abd-Elrazek mahmoud, Basma Mohamed Abd-Elrahman

**Affiliations:** Community Health Nursing Department, Faculty of Nursing, Benha University, Benha, Egypt

**Keywords:** Caregivers, Children, Cerebral palsy, Gross motor skills, Hyperbaric oxygen therapy, Video assisted teaching program, Health care, Medical research

## Abstract

Caregivers play a crucial role in the care of children with cerebral palsy, and their practices greatly influence the development of gross motor skills. A quasi-experimental research design was utilized in the study. This study was conducted in the Hyperbaric Oxygen Therapy Unit at Nasser Institute for Research and Treatment in Cairo Governorate, Egypt. A purposive sample of 76 caregivers and their children with cerebral palsy (age 3–12 years, gross motor level I–III) were assigned to study and control groups. The study group underwent hyperbaric oxygen therapy treatment and received video assisted teaching program for about eight months. Tool I: An interviewing questionnaire comprised of two parts: caregivers- sociodemographic characteristics and children’s personal characteristics and medical history. Tool II: Caregiver Priorities & Child Health Index of Life with Disability to evaluate caregivers practices. Tool III: The Gross Motor Function Measure-88 is used to evaluate changes in the gross motor functions of children with cerebral palsy. The findings revealed that 71.1% and 65.8% of the study and control groups of caregivers were mothers with mean ages of 33.03 ± 4.750 and 32.42 ± 5.006 years, respectively. Before the implementation of the video-assisted teaching program, 34.2% of the children in both the study and control groups exhibited a total gross motor level I. After the program, this percentage increased to 84.2%. The analysis indicated a highly significant positive correlation between the total practices of the studied caregivers and the gross motor skills of their children with cerebral palsy following the implementation of the program. The video-assisted teaching program significantly enhanced the studied caregivers practices regarding care of their children with cerebral palsy, which positively affected gross motor skills among children with cerebral palsy of studied caregivers.

## Introduction

Cerebral palsy (CP), a term used to describe permanent motor and postural developmental impairments, is characterized by limited movement caused by non-progressive brain abnormalities that occur during fetal or early infant development^1^.. It is the most common pediatric disorder that leads to physical disabilities across the nation^2^. Brain damage that results in CP can occur before birth, during delivery, within the first month after birth, or during the early stages of a child’s brain development^3^.

Cerebral palsy is commonly classified based on motor type (spasticity, dyskinesia, and ataxia) and by spastic topography as quadriplegia, diplegia, and hemiplegia. Spastic CP is the most common type, which affects 70–80% of children^4^. The most common risk factors for CP are preterm birth and low birth weight; additional risk factors include multiple pregnancies, hypoxic-ischemic encephalopathy, and maternal infections. The initial brain damage in the majority of CP children’s instances happens during the early stages of fetal brain development^5^. The motor impairments are the most prominent symptoms of CP, often accompanied by disruptions of sensation, perception, cognition, communication, and behavior^6^.

Neurological symptoms and motor impairment are the main criteria used to diagnose CP. Abnormalities found in the brain by Magnetic Resonance Imaging (MRI) can provide insight into the precise date of the causal damage as well as the structural-functional link, and other neurological instruments can be used in diagnosis^7^.

Gross motor skills refer to major abilities like throwing, catching, or hitting items; static balance, like standing/sitting; dynamic balance, as walking; and locomotor skills, like sprinting, jumping, or sliding. The ability of children to walk and carry out daily tasks is referred to as this function^8^. The gross motor skills of children with CP vary greatly; children in level I of the gross motor function are able to walk freely, but those in level V are restricted in their movements and need physical support for all activities^9^.

Several evaluated treatments have demonstrated benefits in enhancing motor function in children with CP. Among these treatments, hyperbaric oxygen therapy (HBOT) is a medical procedure that entails breathing oxygen at different concentrations inside a pressurized chamber^10^. Mechanisms of HBOT for CP are increasing oxygen supply to the brain, improving neurological function, stem cell mobilization, and increasing circulating stem cells. These mechanisms could stimulate cerebral plasticity and lead to motor function improvement^11^ reduce systemic oxidative stress response and increase the expression levels of nerve growth factors in children suffering from cerebral injury^12^.

Community health nurses (CHNs) had a crucial role in the appropriate care of children to overcome or prevent deficiency in children’s care. CHNs and caregivers of children with CP take on a variety of responsibilities, including managing the child’s medical condition, rehabilitation, and meeting needs, in an effort to offer the children the best care possible, which realizes a high standard of living. It’s crucial for CHNs to be included in the therapeutic journey of children with CP. Therefore, evidence-based practice is desperately needed to achieve effective progress and recovery^13,14^.

Video is a technique that uses electronic means to capture, record, store, transmit, and rebuild a series of images that depict moving scenes. Video aids in overcoming language problems because pictures speak without using words^15^. A caregiver video assisted teaching program created specially to support caregivers of children with CP can greatly improve the standard of care and the general health of the caregiver. It enables caregivers of children with CP to offer more efficient, compassionate, and customized assistance, improving the general development and quality of life of their children^16^.

### Significance of the study

Cerebral palsy is the main cause of disability in children. The estimated prevalence in Low-Middle Income Countries (LMICs) is 2–6 cases per 1,000 live births. It is the most prevalent neurological condition in children and a common neurodevelopmental disorder, making up about 50.3% of all cases seen in pediatric clinics^17^. In Egypt, the prevalence of CP among children was around 3.06 per 1000 live births^18^. More teaching and training of caregivers of children with CP help to deal with day-to-day care challenges. This study addresses that gap in caregivers by implementing a video-assisted teaching program aimed at equipping caregivers with skills to support children undergoing HBOT. The use of visual, structured educational content is particularly impactful in enhances caregiver understanding, retention, and confidence in applying care practices at home^19^.

### Aim

The current study aimed to evaluate the effectiveness of video assisted teaching program on caregivers practices and gross motor outcome among children with cerebral palsy undergoing hyperbaric oxygen therapy.

**Research hypotheses**:

1-Practices of caregivers regarding care of children with cerebral palsy undergoing hyperbaric oxygen therapy who will receive video assisted teaching program will be improved than those who will not receive the program.

2- Gross motor skills of children with cerebral palsy of those caregivers who will receive video assisted teaching program will be improved than those who will not receive the program.

## Methods

### Design and setting

We conducted a quasi –experimental research design at Hyperbaric Oxygen Therapy Unit in Nasser Institute for Research and Treatment in Cairo Governorate. Egypt, for eight months from the beginning of May 2024 to end of December 2024.

**Sample**: This study was included: A total 76 caregiver and their children diagnosed with CP from total sample 94 caregiver were recruited using a purposive sample, equivalent homogenous group (study and control group, pre& post test was conducted for both groups). The sample size calculated through **Slovin’s formula** n = N/1 + N(e)2 where:

n = sample size.

N = population size.

e = margin of error = 0,05.

n = N/1 + N(e)2 = 94/1 + 94(0.05)2 = 76 children which would be divided into study 38 and control 38 groups according the following inclusion criteria: Child aged from 3:12 > year, children GMFCS I-III, and children recently receiving the hyperbaric oxygen therapy treatment sessions (first 5 session).

**Data collection tools**:

Data were collected using three tools, which include:

### Tool (I): A structured interviewing questionnaire sheet

This tool was designed, adjusted and prepared in an Arabic language by the researchers and reviewed by the supervisors to collect data about characteristics of the studied caregivers and their children after reviewing related literatures. It consisted of two parts:

Part I: **A**. Sociodemographic characteristics of the studied caregivers of children with CP are caregiver of the child, age, educational level, occupation.

**B.** Personal characteristics of children with CP, such as age, gender, and child ranking among siblings.

Part II: **A.** The medical history of the studied children with CP includes the following: Problems during and after labor, health issues experienced by the children, the age at which each child was diagnosed, the type of CP diagnosed, the treatment regimen prescribed for each child, and their GMFM level.

**B.** History of hyperbaric oxygen therapy, as follows: Oxygen pressure during HBOT session, problems experiences by the children during and after session, and improvement the child has.

### Tool (II) caregiver priorities & child health index of life with disability (CPCHILD)

It was used to evaluate caregivers practices regarding care of children with CP undergoing hyperbaric oxygen therapy adopted from^20,21^ and modified by the researchers. It consisted of two parts:

Part I: Caregivers reported practices included breathing exercises, coughing exercises, range-of-motion exercises, and stretching exercises.

Part II: Caregivers observational practices regarding care of CP children undergoing HBOT, which included positioning practices, transferring and mobility practices, comfort and emotional practices, upper and lower limb massage, and communication and social interaction practices, and health issue practices. Pre and post intervention were conducted.

### Scoring system of caregivers practices

A score of ‘1’ for “done” and ‘0’ for “not done” was assigned for each response. The total points of practices were 45 points. The total practices were classified into two levels: a satisfactory level ≥ 60% (≥ 27 points) of the total practices score, while an unsatisfactory level was < 60% (> 27 points).

### Tool (III) the gross motor function measure (GMFM-88)

It is an observational clinical tool to evaluate change in gross motor skills in children with CP as a result of a video-assisted teaching program along with treatment with HBOT it was adopted from^22^, which is divided into five dimensions of gross motor function: (A) lying and rolling, (B) sitting, (C) crawling and kneeling, (D) standing, and (E) walking, running, and jumping.

### Scoring system for GMFM-88

The total score is obtained by averaging the percentage scores across the five dimensions. Each GMFM item had a four-point scoring range from 0 to 3, where 0 indicates that the child “did not initiate the task,” 1 for “initiated the task (completes < 10% of the activity),” 2 for “partially complete” (completes from 10 to 99% of the activity), and 3 for “completed the task (100%).” Then the dimensions of percentage scoring were calculated as follows: Lying & Rolling (Total Dimension A/51) x 100, Sitting (Total Dimension B/60) x 100, Crawling & Kneeling (Total Dimension C/42) x 100, Standing (Total Dimension D/39) x 100, Walking, Running & (Total Dimension E/72) x 100. Based on the total percentage score across the five dimensions, the child was categorized in which level of GMFM as level I (80%-100%), level II (60%-80%), level III (40%-60%), level IV (20%-40%), or level V (0%-20%).

**Administrative design**:

A formal letter from the dean of the Faculty of Nursing at Benha University was submitted to the director of the Nasser Institute for Research and Treatment to take written permission from the hospital to conduct the study. The letter outlined the study title and objectives.

### Ethics approval and consent to participate

This study has been approved by the Scientific Research Ethics Committee, Faculty of Nursing, Benha University, Egypt (Approval No: REC.CHN.P120). All methods used in the study were carried out in accordance with relevant ethical guidelines of Research Ethics Committee, Faculty of Nursing, Benha University. The verbal and written consents were obtained from all studied caregivers and their children before data collection while ensuring complete privacy and total confidentiality. A complete description of the purpose and nature of the study was approached to all caregivers. All caregivers informed that they have the right to withdraw at any time from the study without explanation of their rationale and their data is secured.

### Pilot study

In order to assess the validity and suitability of the research instruments and determine the caregivers time needed to complete the questionnaire, a pilot study comprising 10% of the sample size—eight caregivers and their children with CP undergoing hyperbaric oxygen therapy was conducted during the first 15 days of May 2024. Since the pilot research showed that no significant changes were made to the study instruments, all participants were included in the sample.

### Content validity

A panel of three community health nursing professors from Benha University Faculty of Nursing reviewed the data collection tools to assess clarity, relevance, comprehensiveness and the accuracy of the information and ensure its appropriateness and applicability. Reliability then conducted to confirm validity of the study tools.

### Reliability

According to the Cronbach’s Alpha coefficient test that was employed to determine reliability, the tools included homogenous items, as shown by the tools’ moderate to high reliability.

- Caregiver Priorities & Child Health Index of Life with Disability scale reliability statistics Cronbach’s alpha = 0.782.
- Gross Motor Function Measure scale reliability statistics Cronbach’s alpha = 0.755.

### Field work

To accomplish the study goals the following four phases were chosen: Assessment, planning, implementation, and evaluation. These phases took place from the beginning of May 2024 to the end of December 2024, covering a period of eight months. The researcher visits the previous setting three days/week from 9 Am to 12:00 Pm because the studied caregivers of children with CP were attending the Hyperbaric Oxygen Therapy Unit on these days Tables 1 and 2.

**Table 1:**
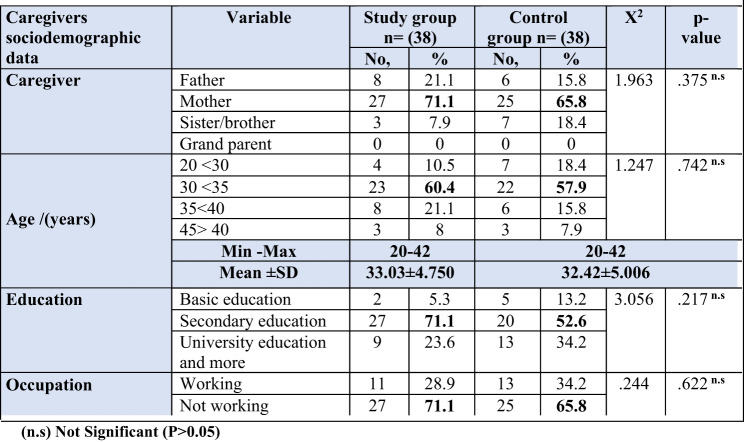
Distribution of both studied caregivers according to their sociodemographic characteristics, study group (n=38), and control groups (n=38)

**Table 2:**
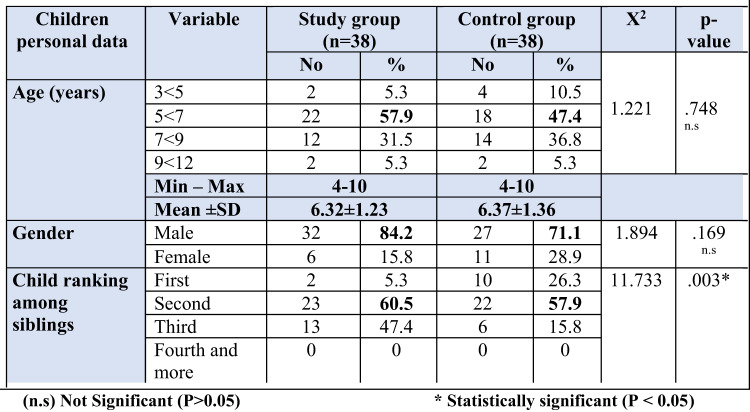
Distribution of both studied children according to their personal characteristics, study group (n=38), and control groups (n=38)

### The program was conducted through

#### A-Assessment phase

Both study and control groups of caregivers were interviewed during the assessment phase to obtain baseline data. The researcher greeted each caregiver and addressed the objectives, timeline, and activities of the study. The researcher was available three days every week (Saturdays, Sundays, and Wednesdays) from 9.00 am to 12.00 pm. The researcher met the caregivers and their children in the waiting area at the Hyperbaric Oxygen Therapy Unit. The data collecting tools were filled during this phase (pretest). The studied caregivers were asked to complete the questionnaire in order to assess caregivers sociodemographic data, children’s personal data and medical and treatment history, practices regarding care of CP children, and gross motor function. Each caregiver was observed individually during their actual practice without observation from caregiver to evaluate their practice exactly using an observational checklist. The average number of caregivers interviewed per day was 3–4 caregivers. The time needed for filling out all data collection tools was 45–60 min. The assessment phase took nearly 8 weeks. Depending on the admitted caregivers who agreed to participate in the study and their response.

**B-Planning phase**:

The researcher designed the video assisted teaching program based on data obtained from the assessment phase and in light of the relevant literature. Accordingly, the researcher used simple Arabic language. Additionally, the researcher prepared and used different teaching methods (lecture, group discussion, demonstration, and re-demonstration) and suitable teaching media (video, and booklet with easy Arabic text and colored pictures) based on caregivers levels of understanding and confirmed the number of sessions. The researcher arranged the time and setting of the sessions with the caregivers. The program objectives were created as follows:

**General objectives**:

The general objective of the video assisted teaching program was to improve studied caregivers practices toward care of their children with CP undergoing HBOT and enhance the children’s GMFs.

**Specific objectives**:

After the completion of the video assisted teaching program, the studied caregivers should be able to:

**Perform the following practical skills**:

Demonstrate steps of breathing and coughing exercises for the child, demonstrate steps of range of motion and stretching exercises for the child, apply steps of positioning and transferring for the child, utilize abilities of each caregiver to promote child comfort, apply steps that help the child improve communication and social interaction, apply steps of ear equalization technique for proper management of the health issue of the child during the HBOT session, and demonstrate and teach steps of finger and upper and lower limb massage to be made for the child during the HBOT session.

**C- Implementation phase**:

The video assisted teaching program was implemented during a period of 4 months (from the beginning of July 2024 to the end of October 2024). The implementation phase included^7^ scheduled sessions. Each session provided for the small group of caregivers, which included 3–4 caregivers, lasted between 45 and 60 min. All groups of studied caregivers receive the same sessions. The program sessions were held in a separate room where the researcher was able to present video and use the different teaching methods. At the beginning of the first session, a brief overview of the video assisted teaching program and its aim was done. Also, the researcher gave the booklet to the study group. Every session started with a brief orientation of the prior one, summary of the previous one and objectives of the new session were provided. The program was implemented through the program sessions. The total number of sessions for the study caregivers was 7 sessions for practical skills, including breathing and coughing exercises, range of motion and stretching exercises, positioning and transferring practices, promoting child comfort practices, promoting child communication and social interaction, ear equalization techniques, and finger and upper and lower limb massage techniques.

At the end of all sessions, the researcher gives the caregivers the opportunity to ask any questions to address their needs and challenges.

**D- Evaluation phase**:

Posttest was done for the study and control groups of caregivers and their children using the same tools that were used in the pretests immediately after the program, aiming to evaluate the efficacy of the video assisted teaching program. The research hypotheses were approved. The evaluation phase took nearly 8 weeks.

#### Statistical analysis

Statistical Package of Social Science (SPSS) version 22 was used to enter and evaluate the data. The methods used for data description are as follows: quantitative data are expressed as mean ± standard deviation. For qualitative data, the chi-square test (X²) was used which displayed as numbers and percent. Spearman analysis was used for correlation analysis. The significance level for statistical tests was set at *P* < 0.05, while the highly significant level was set at *P* < 0.001.

## Results

In the current study Tables 1 and 71.1% and 65.8% of both caregiver groups were mothers, respectively; 60.4% and 57.9% of the study and control groups were aged from 30 years to less than 35 years, with a mean age of 33.03 ± 4.750 and 32.42 ± 5.006 years, respectively; and 71.1% of the study group and 52.6% of the control group had secondary education. and 71.1% and 65.8% of the study and control groups were not working.

Among the enrolled children Tables 2 and 57.9% and 47.4% of them were aged from 5 years to less than 7 years, with a mean age of 6.32 ± 1.23 and 6.37 ± 1.36 years, respectively. Among them, 84.2% and 71.1% were males, and 60.5% and 57.9% of both groups were the second in ranking among siblings, respectively.

In current results Table 3, we found labor hypoxia affected 86.8% of the study group and the control group respectively, and 52.6% of the study group and 39.5% of the control group had fever after labor, respectively. Also, 65.8% and 63.2% of the study group and control group were diagnosed at age > 1 year, respectively, while 78.9% and 73.7% of the study group and 76.3% and 68.4% of the control group were suffering from communication problems and movement problems, respectively. Furthermore, 60.5% of the study group and 71.1% of the control group had GMF level II, respectively.

The results Table 4 show that 65.7% and 71% of both groups received HBOT sessions at 1.5 ATA. During the HBOT session, 78.9% of the study group and 68.4% of the control group experienced severe ear pain, and 89.5% and 81.6 of the study group and control group experienced fatigue following the HBOT session, respectively. Cognitive function and communication skills in the study group increased by 81.6% and 68.4%, respectively, while the control group’s improvements were 78.9% and 68.4%.

**Table 3:**
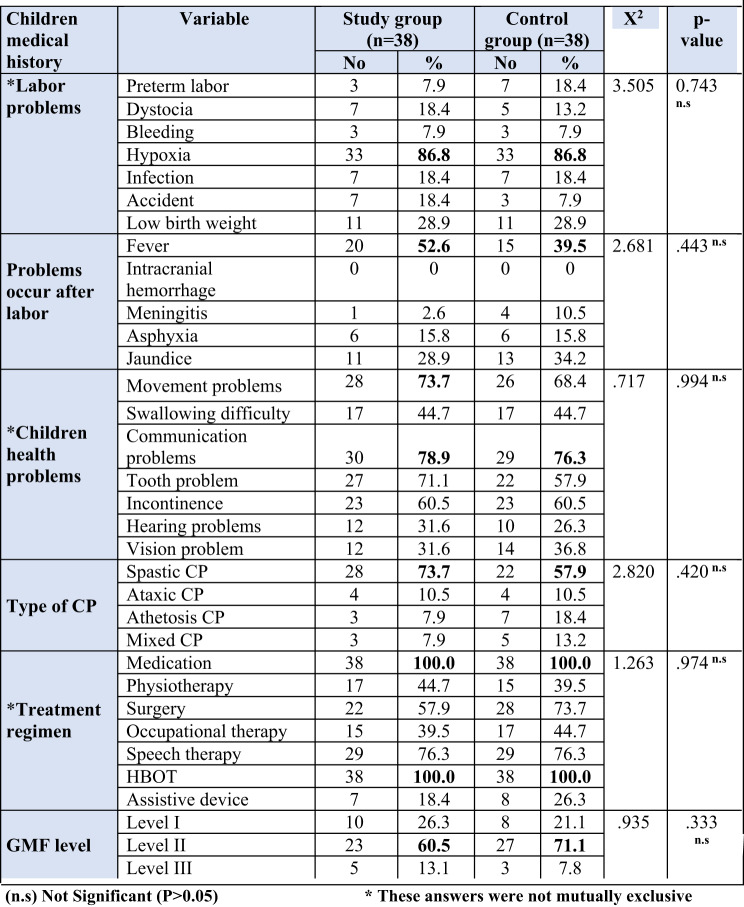
Distribution of both studied children according to their medical history, study group (n=38), and control groups (n=38)

The current results Table 5 show that there was a highly statistically significant difference between the study and control groups regarding total observational practices of positioning, comfort and emotional, limbs massage, and health issue domains post of video assisted teaching program implementation (*p* > 0.001). While, there was statistically significant difference between both groups regarding observational transferring practices and communication and social interaction domains post of video assisted teaching program implementation (*p* > 0.05).

**Fig. 1:**
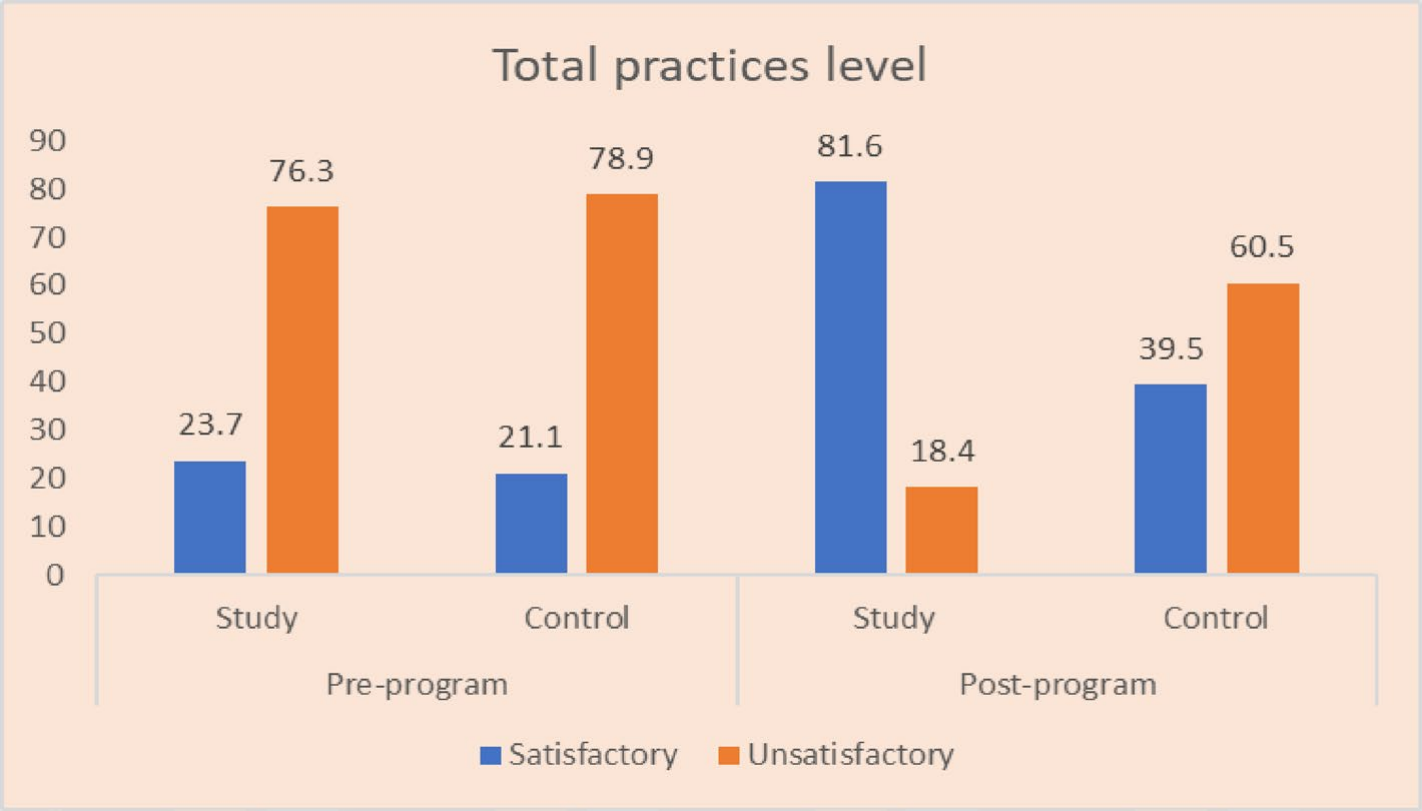
Percentage of both studied caregivers regarding their total practices’ items pre and post program, study group (*n* = 38) and control groups (*n* = 38).

Before the implementation of the teaching program, 23.7% of the study group demonstrated a satisfactory level of total practices. This percentage markedly increased to 81.6% after the program Fig. 1. In contrast, 21.1% of the control group had a satisfactory level of total observational practices at the pre-program, which slightly rose to 39.5% at the post teaching program.

**Fig. 2:**
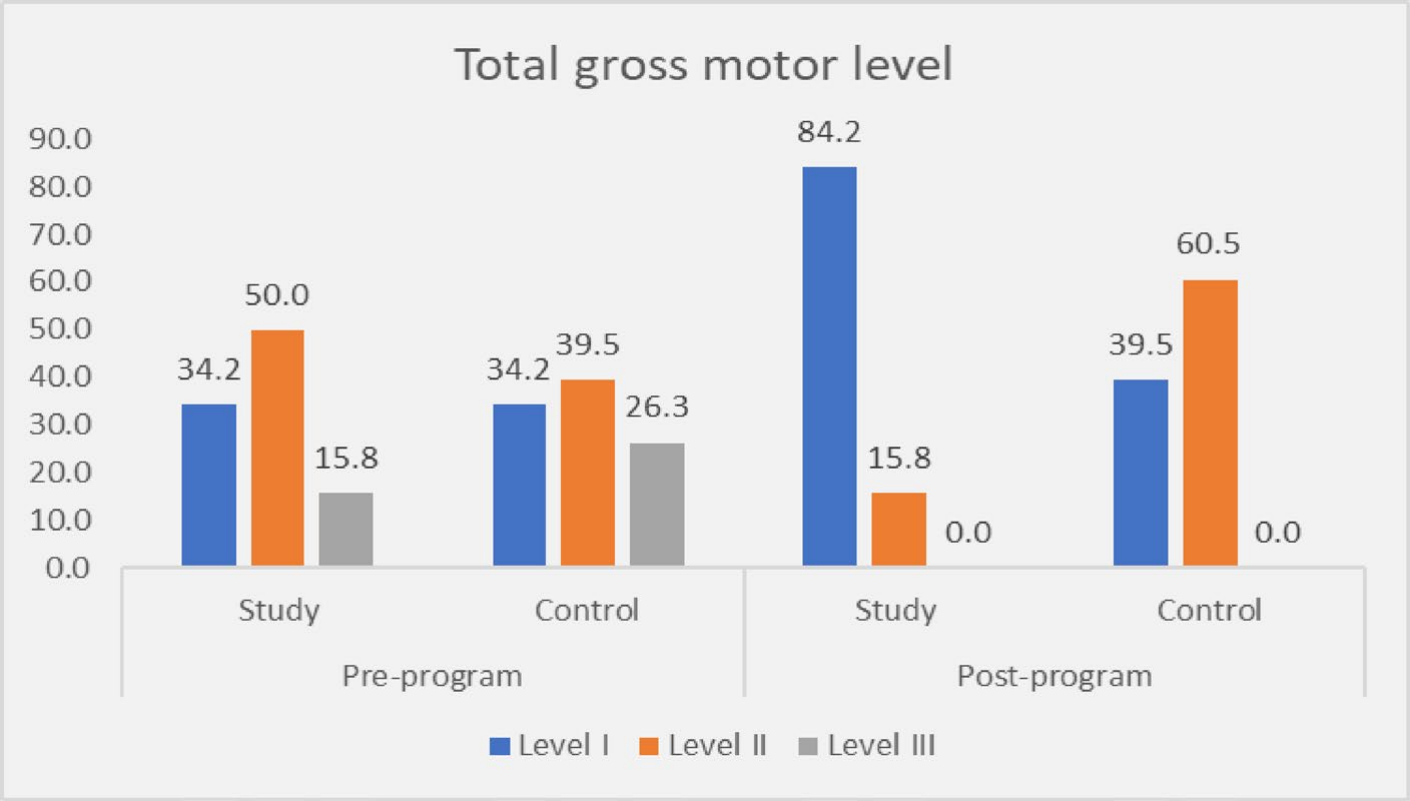
Percentage of studied children regarding their total level of gross motor function pre and post program, study group (*n* = 38) and control groups (*n* = 38).

Total gross motor level I of the study group increased from 34.2% pre teaching program to 84.2% post teaching program respectively Fig. 2. Conversely, 34.2% of the control group had a total gross motor level I pre teaching program elevated to 39.5% post teaching program, respectively.

Owing to substantial correlation between total gross motor function of studied children and total practices of studied caregivers Table 6 there was a statistical moderate positive correlation between total gross motor function of both studied children and total practices of both groups pre video assisted teaching program implementation (*P* > 0.05 & *r* = 0.453), and was changed to a statistical strong positive post video assisted teaching program implementation (*P* > 0.001 & *r* = 0.747).

**Table 4:**
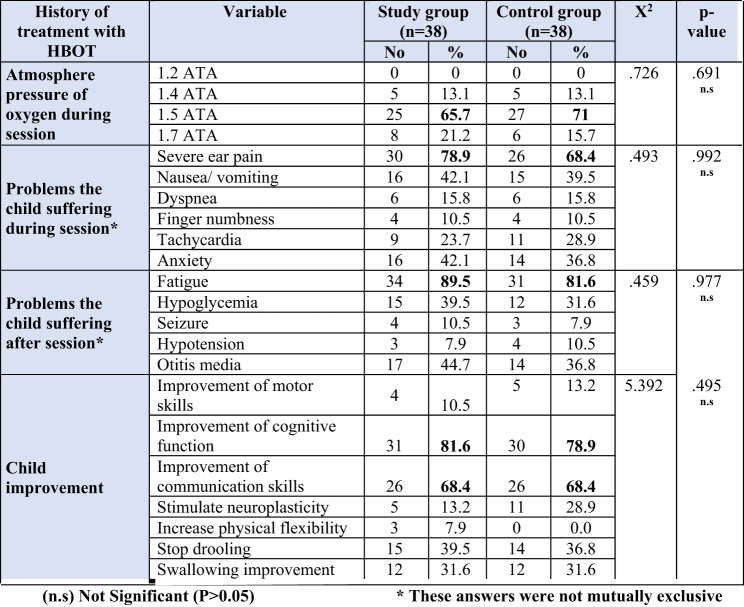
Distribution of studied children according to their history of treatment with HBOT, study group (n=38), and control groups (n=38)

**Table 5:**
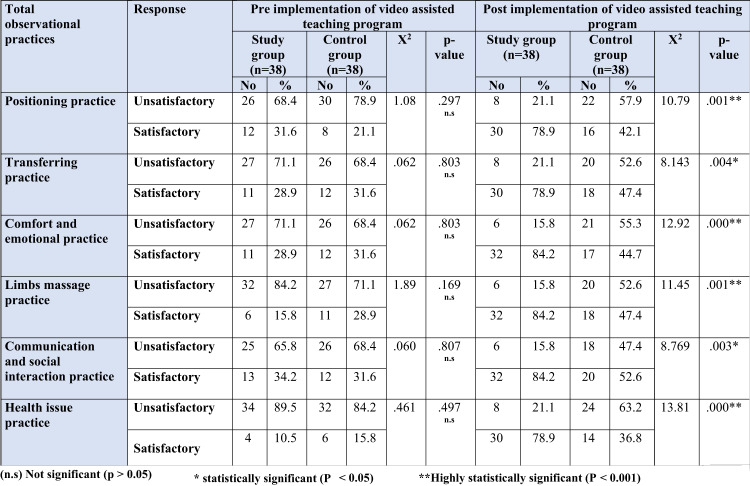
Comparison of both studied groups of caregivers regarding their total observational practices pre and post program, study group (n=38) and control groups (n=38).

**Table 6:**
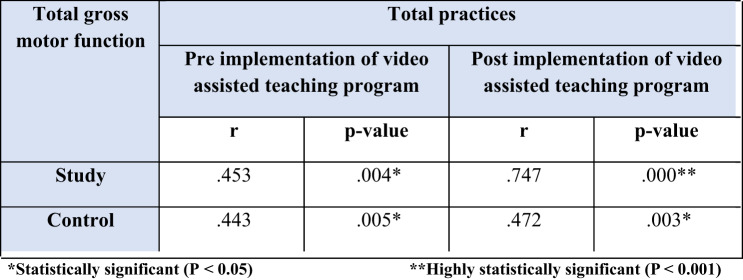
Correlation between total gross motor function score of studied children and total practices of caregivers among study and control groups pre and post program.

## Results

### Discussion

Regarding sociodemographic characteristics of studied caregivers Table (1); the present study displayed that three fifths (60.4%) and lightly less than three fifths(57.9%) of the study and control groups aged from 30 years to less than 35 years with mean age 33.03 ± 4.750 and 32.42 ± 5.006 years respectively, While, less than three quarters (71.1%) of the study group and 52.6% of the control group had secondary education. While, less than three quarters (71.1%) and almost two thirds (65.8%) of the study and control groups were not working.

Concerning personal data of the studied children Table (2), the present study indicated that more than half (57.9%) and approximately less than half (47.4) % of the study and control groups aged from 5 years to less than 7 years with mean age 6.32 ± 1.23 and 6.37 ± 1.36 years respectively. Also, majority (84.2%) of the study group and less than three quarters (71.1%) of control group were males, and three fifths (60.5%) and more than half (57.9%) of the study and control groups were the second in ranking among siblings respectively.

According to Table (3), which details the medical history of the studied children, the majority of the study group and control group (86.8%) had labor hypoxia. This conclusion was supported by^23^, who discovered that issues throughout pregnancy and hypoxia during labor were the primary causes of cerebral palsy. This is also inconsistent with^24^, who found that prenatal hypoxia affects less than two-fifths (37.1%) of the sample. Additionally, the study concerning the features of epilepsy in children with cerebral palsy indicated that less than one-third (29.3%) experience birth asphyxia, which contradicted this finding^25^. This might be the result of oxygen-marked newborn hypoxia which puts the baby at serious danger for brain hypoxia and acidosis because of the high level of carbon dioxide in the blood, which leads to ischemic encephalopathy, which can result in brain injury, which is considered one of the main causes of newborn death or severe postnatal neurological disorders, including cerebral palsy.

Additionally, the current study determined that fever after labor was experienced by nearly two-fifths (39.5%) of the control group and more than half (52.6%) of the study group. According to a study on the clinical profile and related comorbidities of cerebral palsy in children, fever accounted for almost a quarter (26.2%) of postnatal risk factors. This finding was consistent with that of^26^. Disagreed with^27^, who discovered that 25.45% of the study sample suffered from postnatal fever. This might be the result of an infection, which triggers an immunological reaction that results in the production of proteins known as cytokines, which in turn induce inflammation and brain damage.

Furthermore, the current investigation, less than three quarters (73.7%) of the study group and more than two thirds (68.4%) of the control group, respectively, had movement issues. This result was consistent with research by^28^, which examined the impact of family empowerment programs on parents’ performance in caring for children with cerebral palsy and found that almost two-thirds (69.1%) of the children in the study had movement issues. This might be because CP damages or malforms the parts of the brain that regulate motor function, which interferes with the brain’s capacity to regulate movement and impairs a child’s muscle coordination.

Regarding history of treatment with HBOT of studied children; in the current study the majority of the study group (81.6%) and more than three quarters (78.9%) of the control group had improved cognitive performance. These results are consistent with^29^, who reported that the general cognitive score was significantly improved in the HBOT with a mean score improved (90.2 ± 10.3 to 96.1 ± 10.3, *p* = 0.01) compared to the control group (95.1 ± 7.5 to 94.9 ± 7.7, *p* = 0.96). The study was conducted in Saudi Arabia and involved 52 children. This might be HBOT improved the microstructure of the brain in specific cortical areas, which are linked to improvement in cognitive function, such as verbal fluency, working memory, and executive function.

Regarding total observational practices of both studied caregivers Table (5); the current study shows that there was a highly statistically significant difference between the study and control groups regarding total observational practices of positioning, comfort and emotional, limb massage, and health issue domains post of video assisted program. This finding was parallel with^30^, who concluded that the total practice score of the study ‘group demonstrated highly statistical differences at (F = 129.9, P-value ≤ 0.001, ɳ² = 0.76), in contrast to the control group results, which showed insignificant differences at (F = 3.96, P-value = 0.054, ɳ² = 0.09). This might be due to teaching programs to teach caregivers key principles related to safe, effective practices and provide opportunities for demonstrations and redemonstrations that enhanced caregivers knowledge, skills, confidence, consistency, and motivation—leading to measurable improvements across all observed caregiving tasks.

Corresponding caregivers total practices level figure (1); the existing study showed that more than one-fifth (23.7) of the study group had a satisfactory total practices level pre-teaching program. While the majority (81.6%) of the study group had a satisfactory total practices level post-teaching program. This finding was in the same line with^31^, who said that total practice, less than one-fifth (13.3%) of studied caregivers had satisfactory practice pre-program compared to most (90.7%) of them post-program, with a highly statistically significant difference pre- and post-program at (p-value < 0.001). This might be due to use a simple Arabic language during the teaching program and used illustrative methods as videos and photos which enable caregivers to overcome their challenges and enhancing child care practices. This finding supports hypothesis No. 1.

Concerning the total gross motor level among both studied children figure (2); the current study illustrated that the total gross motor level I of the study group increased from a third (34.2%) pre-teaching program to a majority (84.2%) post-teaching program, respectively. While a third (34.2%) of the control group had a total gross motor level of I pre-teaching program, it improved to two-fifths (39.5%) post-teaching program. These findings were parallel with^32^, who revealed that there was a significant increase in GMFM scores and a decrease in level post-intervention in both groups (*p* < 0.05). Higher scores indicate improvement in gross motor function, respectively. This might be due to training programs equipping caregivers with specific skills that promote motor skills development. This finding supports hypothesis No. 2.

The results of the current study Table (6); indicated that there was a significant positive correlation between total practices of caregivers and total gross motor function of children in the study and control groups pre- and post-video-assisted teaching program implementation (*p* ≤ 0.001), respectively. This finding parallel^33^, who performed a study regarding the relationship between gross motor function ability and time use in mothers of children with cerebral palsy and found that mothers activities have significant correlations with gross motor function abilities of their children (*P* < 0.05). Also supported by^34^, who said that there is a relationship between the mother’s role and motor development in children (p value < 0.001). From the researcher’s point of view, this might be a caregiver who implements consistent intervention, as physical exercises and daily routines can help to improve motor skills in children with CP.

### Limitations of the study

A few limitations applied to this study: Crowding at hyperbaric units and crying of children were limitation for researcher and some session took longer than expected due to frequent interruption.

## Conclusion

The research hypotheses were validated based on the results of the current investigation. Compared to before the teaching program was implemented, the improved caregivers practices results on improved the gross motor functions of their children.

### Recommendation

Based on finding of present study the following recommendations are suggested:

- Developing web page for caregivers of children with CP to supply with scientific health information regarding care of children undergoing hyperbaric oxygen therapy.
- Continuous educational program for caregivers to improve practices regarding care of CP children during hyperbaric oxygen therapy session.

## Data Availability

The data that support the findings of this study are available on request from the corresponding author.
